# Metabolic Value Chemoattractants Are Preferentially Recognized at Broad Ligand Range Chemoreceptor of *Pseudomonas putida* KT2440

**DOI:** 10.3389/fmicb.2017.00990

**Published:** 2017-05-31

**Authors:** Matilde Fernández, Miguel A. Matilla, Álvaro Ortega, Tino Krell

**Affiliations:** Estación Experimental del Zaidín, Department of Environmental Protection, Consejo Superior de Investigaciones Científicas,Granada, Spain

**Keywords:** chemotaxis, chemoreceptor, ligand recognition, isothermal titration calorimetry, *Pseudomonas*

## Abstract

Bacteria have evolved a wide range of chemoreceptors with different ligand specificities. Typically, chemoreceptors bind ligands with elevated specificity and ligands serve as growth substrates. However, there is a chemoreceptor family that has a broad ligand specificity including many compounds that are not of metabolic value. To advance the understanding of this family, we have used the PcaY_PP (PP2643) chemoreceptor of *Pseudomonas putida* KT2440 as a model. Using Isothermal Titration Calorimetry we showed here that the recombinant ligand binding domain (LBD) of PcaY_PP recognizes 17 different C6-ring containing carboxylic acids with *K*_D_ values between 3.7 and 138 μM and chemoeffector affinity correlated with the magnitude of the chemotactic response. Mutation of the *pcaY_PP* gene abolished chemotaxis to these compounds; phenotype that was restored following gene complementation. Growth experiments using PcaY_PP ligands as sole C-sources revealed functional relationships between their metabolic potential and affinity for the chemoreceptor. Thus, only 7 PcaY_PP ligands supported growth and their *K*_D_ values correlated with the length of the bacterial lag phase. Furthermore, PcaY_PP ligands that did not support growth had significantly higher *K*_D_ values than those that did. The receptor has thus binds preferentially compounds that serve as C-sources and amongst them those that rapidly promote growth. Tightest binding compounds were quinate, shikimate, 3-dehydroshikimate and protocatechuate, which are at the interception of the biosynthetic shikimate and catabolic quinate pathways. Analytical ultracentrifugation studies showed that ligand free PcaY_PP-LBD is present in a monomer-dimer equilibrium (*K*_D_ = 57.5 μM). Ligand binding caused a complete shift to the dimeric state, which appears to be a general feature of four-helix bundle LBDs. This study indicates that the metabolic potential of compounds is an important parameter in the molecular recognition by broad ligand range chemoreceptors.

## Introduction

Bacteria need to constantly adapt to changing environmental conditions to assure survival. These adaptations are achieved through a variety of signal transduction systems that most commonly include one- and two-component systems as well as chemosensory pathways (Galperin, 2005; Laub and Goulian, 2007; Hazelbauer et al., 2008). In the latter systems, signaling is initiated either by the direct binding of chemoeffectors or chemoeffector-loaded periplasmic binding proteins to the ligand binding domain (LBD) of chemoreceptors (Matilla and Krell, 2017). Chemoreceptor activation triggers a molecular stimulus that modulates CheA autophosphorylation and consequently transphosphorylation of the CheY response regulator, which generates the pathway output (Hazelbauer et al., 2008). Chemosensory pathways were shown to mediate chemotaxis and type IV pili-based motility, or are involved in regulating alternative cellular processes (Hickman et al., 2005; Zusman et al., 2007; Wuichet and Zhulin, 2010).

Chemotaxis provides bacteria with the capacity to approach or escape from different compounds, therefore favoring the movement toward optimal ecological niches. Thus, chemotaxis systems of different bacteria have evolved to facilitate adaptation to specific ecological niches. *Escherichia coli* is the traditional model to study chemosensory signaling mechanisms. This bacterium has four chemoreceptors and an aerotaxis receptor that feed into a single chemotaxis pathway (Parkinson et al., 2015). However, several alternative model organisms have been established to investigate chemotaxis in bacteria with lifestyles different to that of the enterobacterium *E. coli*.

One of these models is *Pseudomonas putida* KT2440 (Belda et al., 2016). This strain was isolated from soil, is nutritionally versatile, has a saprophytic lifestyle, colonizes plant roots efficiently and is able to protect plants against phytopathogens (Bagdasarian et al., 1981; Espinosa-Urgel et al., 2002; Nakazawa, 2002; Regenhardt et al., 2002; Matilla et al., 2010). *P. putida* KT2440 has 27 different chemoreceptors and data available suggest that receptors feed into three chemosensory pathways that are homologous to the *che*, *wsp*, and *chp* pathways of *P. aeruginosa* (Garcia-Fontana et al., 2013). Information on the function of many chemoreceptors of *P. putida* KT2440 has been obtained which are summarized in Supplementary Figure 1 and Table 1. Characterized KT2440 chemoreceptors include McpS (Lacal et al., 2010; Pineda-Molina et al., 2012), McpQ (Martin-Mora et al., 2016) and McpR (Parales et al., 2013), for different tricarboxylic acid (TCA) cycle intermediates, McpP for C2- and C3-organic acids (Garcia et al., 2015), McpA for L-amino acids (Corral-Lugo et al., 2016), McpU for polyamines (Corral-Lugo et al., 2016), McpG for gamma-aminobutyrate (Reyes-Darias et al., 2015a) or McpH for metabolizable purines (Fernandez et al., 2016). In addition, there are three paralogous receptors for energy taxis (Sarand et al., 2008) as well as homologs of the *P. aeruginosa* WspA and PilJ chemoreceptors, that feed into chemosensory pathways regulating c-di-GMP levels or mediate type IV pili-based motility, respectively (Hickman et al., 2005; Persat et al., 2015; Corral-Lugo et al., 2016). The remaining 14 chemoreceptors are of unknown function.

The inspection of ligand profiles of chemoreceptors suggests that the primary physiological function of chemotaxis resides in the capacity to approach compounds that are of metabolic value and that can be used for growth. For example, all 34 compounds that were shown to bind and activate the eight characterized KT2440 chemoreceptors mentioned above serve the bacterium either as carbon or nitrogen sources. However, there is evidence for another class of chemoreceptors that is characterized by a very broad ligand range and where only few of the recognized chemoattractants appear to be of metabolic value. One such chemoreceptor is McpT of *P. putida* DOT-T1E (Lacal et al., 2011), which recognizes and mediates chemotaxis to a wide range of different mono- and biaromatic compounds of which only few can be used as nutrient sources. The physiological sense of the response to the remaining compounds is unclear, but may represent the sensing of certain environmental cues as proposed by Yang et al. (2015).

Another member of this broad ligand range receptor family is PcaY of *P. putida* F1; a chemoreceptor that responds to a number of C6-ring containing carboxylic acids (Luu et al., 2015). PcaY chemoeffectors include, for example, the non-aromatic quinate and shikimate as well as various aromatics like benzoate, 4-hydroxybenzoate, protocatechuate, vanillate and vanillin. Interestingly, Luu et al. (2015) observed weak sequence similarities (16% identity) between the LBDs of McpT and PcaY. As for the mode of chemoeffector recognition, the authors expected that PcaY binds aromatic acids, but were unable to rule out indirect binding mediated by periplasmic binding proteins. In addition, it could not be ruled out that downstream intermediates are sensed by the chemoreceptor rather than the compounds themselves (Luu et al., 2015). Precedents for such mechanism have recently been reported for the chemoreceptors MCP2201 and MCP2983 of *Comamonas testosteroni* that mediate chemotaxis to multiple aromatic compounds, including several PcaY effectors such as 4-hydroxybenzoate, vanillate, vanillin, and protocatechuate (Ni et al., 2013a, 2015). The authors showed that none of these compounds bound to the recombinant LBDs of these receptors (Ni et al., 2013a, 2015). Instead, different TCA cycle intermediates bound and it was proposed that chemotaxis toward aromatics is caused by the recognition of compounds that are generated during aromatic compound metabolism.

The ORF encoding the chemoreceptor PP2643 of *P. putida* KT2440 is homologous to PcaY of *P. putida* F1 (97% sequence identity) and has therefore been named PcaY_PP. In this study, we used PcaY_PP as a model to study ligand recognition at broad ligand range chemoreceptors. We will define the ligand profile of this receptor, assess whether receptor activation occurs by direct binding or an alternative indirect mechanism and evaluate the relationship between the receptor affinity and metabolic value of the different ligands. Our data show that this chemoreceptor recognizes preferentially ligands that are of metabolic value. A significant number of ligands were of no apparent metabolic value and may correspond to environmental cues that are sensed by the bacterium.

## Experimental Procedures

### Bacterial Strains, Culture Media, and Growth Conditions

Bacterial strains used in this study are listed in **Table 1**. *P. putida* KT2440 and its derivative strains were routinely grown at 30°C in LB or M9 medium supplemented with 1 mM MgSO_4_, 6 mg l^-1^ Fe-citrate (Abril et al., 1989) and 10 mM sodium benzoate or glucose as carbon source. *P. putida* KT2440R was grown in the same medium supplemented with rifampicin (10 μg ml^-1^). For growth experiments to assess the capacity of the PcaY_PP ligands to support growth a sole C-source, KT2440 cells were pre-cultured overnight in M9 medium supplemented with 10 mM glucose and washed twice with M9 medium, prior to the inoculation of M9 medium containing 10 mM of the different carbon sources; except for 3-CBA, 4-CBA, 3-NBA, 4-NBA, 3-MBA, and 4-MBA that, due to their lower solubility, were used at 2 mM. When necessary, the pH of the medium was adjusted to 7.0 prior to inoculation. Bacterial growth rates were monitored by measuring the OD_660_ until cultures reached the stationary phase. The growth rate during the exponential growth (m) was calculated according to m = (LnOD_2_-LnOD_1_).(t_2_-t_1_)^-1^, where OD_2_/t_2_ and OD_1_/t_1_ correspond to the OD_660_/time at the end and the beginning of the time interval. The generation time (G) was defined by *G* = Ln2 μ^-1^. The duration of the lag phase was calculated from the moment of inoculation to the start of the exponential phase as described in Swinnen et al. (2004). Normalized growth yield per carbon source after 24 h was determined applying correlation factors between cell dry weight and OD_660_ determined in batch cultures and assuming that all the carbon source was consumed during growth. Data were fitted by linear least squares regression or submitted to the chi-squared test with Yates correction using in both cases the Graph Pad Prism v7.02 software.

**Table 1:** Bacterial strains and plasmids used in this study.

Strain or plasmid	Relevant characteristics^a^	Reference
**Strains**		
*Escherichia coli* BL21 (DE3)	F^-^, *ompI, hsdS*_B_ (r^-^_B_ m^-^_B_) *gal*, *dam*, *met*	Jeong et al., 2009
*E. coli* DH5α	*supE44 lacU169* (*Ø80lacZ*ΔM15) *hsdR17* (*r_k_*^-^*m_k_*^-^), *recA1 endA1 gyrA96 thi-1 relA1*	Woodcock et al., 1989
*Pseudomonas putida* KT2440	Wild type	Belda et al., 2016
KT2440R	Rif^R^ derivative of *P. putida* KT2440; wild type	Espinosa-Urgel and Ramos, 2004
KT2440RTn*7*-ΩSm1	Rif^R^, Sm^R^; extragenic site-specific insertion of mini-Tn*7*	Matilla et al., 2007
KT-PcaY	Rif^R^, Km^R^; *P. putida* KT2440R transposon mutant *pp2643*::mini-tn*5-*ΩKm.	Duque et al., 2007
**Plasmids**		
pET28b(+)	Km^R^; Protein expression plasmid	Novagen
pBBR1MCS-5	Gm^R^; *oriRK2 mobRK2*	Kovach et al., 1995
pET28-PcaY_PP-LBD	Km^R^; pET28b(+) derivative containing DNA fragment encoding PcaY_PP-LBD	This study
pMAMV260	Gm^R^; pBBR1MCS-5 derivative containing *pcaY_PP* gene and its 482 bp upstream region	This study

*^a^ Km, kanamycin; Rif, rifampin; Gm, gentamicin.*

### Construction of the Expression Plasmid for PcaY_PP-LBD

The PcaY_PP-LBD (amino acids 44–196) was defined as the segment between both transmembrane regions as predicted by the DAS transmembrane region prediction algorithm (Cserzo et al., 1997). The corresponding DNA fragment was amplified by PCR using primers 5′-AACATATGGGTAGCGACCAGCAGATCAC-3′, containing a NdeI restriction site (underlined), and 5′-AAGGATCCCTACCGGGCCAGACGCCGGTCGGA-3′, containing a BamHI site (underlined) as well as a stop codon. The PCR product was cloned into pET28b (Novagen), previously digested with the same enzymes. The ligation mixture was dialyzed and electroporated into *E. coli* DH5α. Transformants were selected on kanamycin (50 μg ml^-1^) LB plates and the resulting plasmid pET28-PcaY_PP-LBD was verified by sequencing the insert and flanking regions prior to the transfer into *E. coli* BL21 (DE3) by electroporation.

### Protein Overexpression and Purification

*Escherichia coli* strain BL21 (DE3) pET28-PcaY_PP-LBD was grown in 2 l Erlenmeyer flasks containing 500 ml LB supplemented with 50 μg/ml kanamycin at 30°C until an OD_660_ of 0.6, at which point protein expression was induced by adding 0.5 mM isopropyl-beta-D-thiogalactopyranoside. Growth was continued at 18°C overnight and cells were harvested by centrifugation at 10,000 *g* for 30 min. Cells were then resuspended in buffer A (20 mM Tris/HCl, 200 mM NaCl, 10 mM imidazole, 5 % (v/v) glycerol, 0.1 mM EDTA, pH 8.0) and broken by French Press treatment. After centrifugation at 20,000 g for 15 min, the supernatant was loaded onto a 5 ml HisTrap column (Amersham Bioscience), washed with 10 column volumes of buffer A and eluted with an imidazole gradient of 45–500 mM in the same buffer. Protein containing fractions were pooled and dialyzed overnight at 4°C into analysis buffer (5 mM PIPES, 5 mM MES, 5 mM Tris-HCl, pH 8.0).

### Isothermal Titration Calorimetry (ITC)

Experiments were carried out at 25°C using a VP-ITC titration calorimeter (Microcal Inc., Northampton, Massachusetts). Protein at 25 μM in analysis buffer was degassed prior to its introduction into the sample cell and titrated with aliquots of 500 μM to 3 mM ligand solutions prepared in the same buffer. Obtained data were corrected with the heat changes measured for the injection of ligand into buffer. Data were normalized with the protein concentration (Bradford assay) and fitted with the “One Binding Site” model of the MicroCal version of the ORIGIN 7.0 software (OriginLab Corporation, Northampton, MA, United States), leaving all parameters floating.

### Analytical Ultracentrifugation

Experiments were performed in a Beckman Coulter Optima XL-A analytical ultracentrifuge (Beckman-Coulter, Palo Alto, CA, United States) equipped with UV-visible absorbance detection system, using an An50Ti 8-hole rotor, 12 mm path-length charcoal-filled epon double-sector centerpieces. The sedimentation velocity (SV) experiments were carried out at a rotor speed of 40,000 rpm and 10°C using 400 μl samples in analysis buffer. Protein was at 15–280 μM whereas shikimate, protocatechuate and quinate were added, when indicated, at final concentrations of 400 μM. Analysis buffer with or without ligands was used as reference. Measurements were made at 232 nm in the absorbance optics mode. A least squares boundary modeling of the SV data was used to calculate sedimentation coefficient distributions with the size-distribution c(s) method (Schuck, 2000) implemented in the SEDFIT v11.71 software. Buffer density (ρ = 1.00025 g/ml) and viscosity (η = 0.01314 Poise) at 10°C were calculated from the buffer composition using SEDNTERP software (Laue et al., 1992). The partial specific volume (0.7139 ml/g) and the monomeric molecular weight (19.2 kDa) of PcaY_PP-LBD were also calculated from its amino acid sequence using this software. The multi-speed sedimentation equilibrium experiments were carried out at 10°C and 12,000, 14,000, and 18,000 rpm until equilibrium was reached. Sedimentation was followed by measuring absorbance at 235, 240, 280, and 290 nm depending on the sample concentration. Proteins were measured at six concentrations ranging from 15 to 280 μM. Generally two wavelengths were used per sample. A monomer–dimer self-association model implemented in SEDPHAT (Vistica et al., 2004) was used for a global fit that accounts for all concentrations, speeds and wavelength.

### Construction of Plasmid for Genetic Complementation Assays

For the construction of the complementing plasmid, the *pcaY_PP* gene and its promoter region were amplified by PCR using primers 5′-TAATAAGCTTCGGCGAACAGATCAGCGTG-3′ and 5′-TAATGGATCCGCAGAGGTCAGGCAGCGACG-3′. The resulting fragment was digested with HindIII and BamHI and cloned into the same sites in pBBR1MCS-5 to generate pMAMV260. The insert was confirmed by PCR and sequencing, and pMAMV260 was used to transform the *pcaY_PP* defective mutant by electroporation.

### Quantitative Capillary Chemotaxis Assays

Overnight cultures of *P. putida* KT2440 strains were diluted to an OD_660_ of 0.05 in M9 minimal supplemented with 10 mM benzoate as carbon source and grown at 30°C with orbital shaking (200 rpm). At an OD_660_ of ∼0.4 (early stationary phase of growth), the cultures were centrifuged at 1,700 *g* for 5 min and the resulting pellet was washed twice with chemotaxis buffer (50 mM potassium phosphate, 20 μM EDTA, 0.05% (v/v) glycerol, pH 7.0). Subsequently, the cells were re-suspended in the same buffer, adjusted to an OD_660_ of 0.15 and 230 μl aliquots of this bacterial culture were placed into 96-well plates. For the quantitative assays, one-microliter capillary tubes (Microcaps, Drummond Scientific, Ref. P1424) were heat-sealed at one end and filled with either the chemotaxis buffer (negative control) or chemotaxis buffer containing the chemoeffector. The capillaries were immersed into the bacterial suspension and incubated for 30 min at room temperature. Capillaries were subsequently removed from the bacterial suspension, rinsed with sterile water and the content expelled into 1 ml of M9 medium. Serial dilutions were plated onto LB medium (containing the appropriate antibiotics) and the number of colony forming units was determined. In all cases, data were corrected with the number of cells that swam into buffer containing capillaries.

### Competitive Root Colonization Assays

Sterilization, germination and inoculation of maize seeds was carried out as described previously, with minor modifications (Matilla et al., 2007). Briefly, sterile seeds were incubated for 30 h at 30°C with a 5 × 10^6^ CFU/ml 1:1 mixture of KT2440RTn*7*-ΩSm1 (wild type) and KT-PcaY (mutant). Thereafter, seeds were rinsed with sterile deionized water and planted in 50 ml Sterilin tubes containing 40 g of sterile washed silica sand and 10% (v/w) plant nutrient solution (5 mM Ca(NO_3_)_2,_ 5 mM KNO_3,_ 1 mM MgSO_4_, 0.5 mM KH_2_PO_4_, pH 7.0) supplemented with Fe-EDTA (100 μM) and micronutrients. Plants were maintained at 24°C with a daily light period of 16 h. After 7 days, bacterial cells were recovered from the rhizosphere or from 1 mm of the main root apex, as described previously (Matilla et al., 2007). Serial dilutions were plated on LB-agar medium supplemented with rifampicin and streptomycin (or kanamycin) to select KT2440RTn*7*-ΩSm1 or the *pcaY_PP* mutant strain, respectively.

## Results

### PcaY_PP Recognizes Aromatic and Non-aromatic Chemoeffectors Directly

**FIGURE 1:**
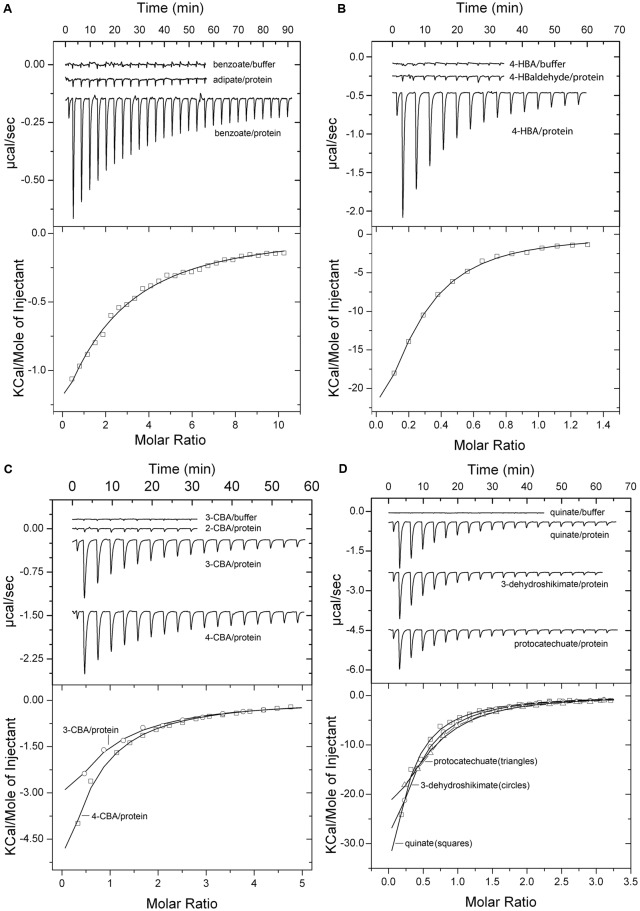
Microcalorimetric studies of the binding of different ligands to purified PcaY_PP-LBD. Upper panels: raw titration data; the upper traces correspond to the injection of ligand into buffer (control). Lower panels: Integrated, concentration-normalized and dilution heat corrected peak areas of the raw data. The line is the best fit to the “One binding site model” of the MicroCal version of ORIGIN. Protein concentration was of 25 μM, injection volumes were between 4.8 and 9.6 μl and ligand concentrations between 0.5 and 2 mM. Titration with benzoate and adipate **(A)**; 4-hydroxybenzoate and 4-hydroxybenzaldehyde **(B)**; 2-chlorobenzoate, 3-chlorobenzoate and 4-chlorobenzoate **(C)**; quinate, 3-dehydroshikimate and protocatechuate **(D)**.

**FIGURE 2:**
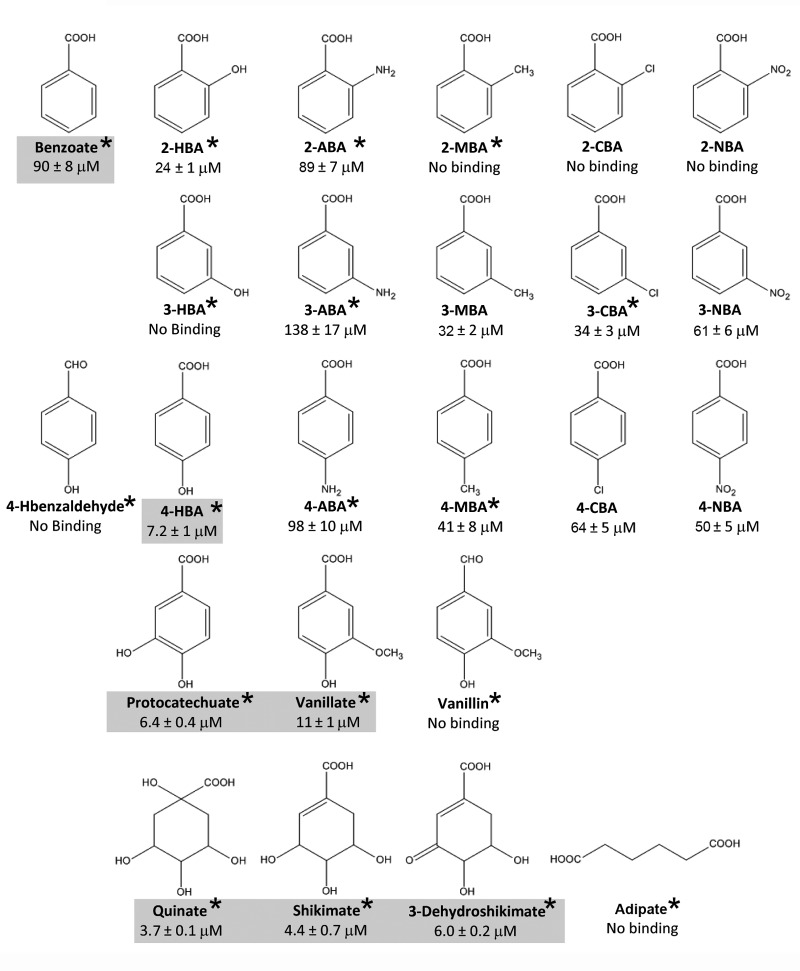
Summary of results from microcalorimetric titrations of PcaY_PP-LBD with different ligands. Shown are structures and the corresponding *K*_D_ values as determined by ITC. The gray shading indicates the capacity to support growth of KT2440 as sole carbon source. Data are means and standard deviations from three experiments. Asterisks indicate that it is a natural compound according to the Zinc database (Irwin et al., 2012). For further information on these compounds see Supplementary Table 3.

The initial objective in the study of PcaY_PP was to identify the mode of ligand recognition. Previous research has shown that most individual chemoreceptor LBDs fold into stable domains that bind their chemoeffectors with the same affinity as the full-length receptor (Milligan and Koshland, 1993; Matilla and Krell, 2017). To identify the LBD of PcaY_PP (PcaY_PP-LBD), its sequence was analyzed by the DAS transmembrane prediction server (Cserzo et al., 1997) and the DNA fragment encoding the segment between both transmembrane regions, predicted to be four-helix bundle domain, was cloned into an expression vector. The protein was overexpressed in *E. coli* and purified from the soluble fraction of its cell lysate. Subsequently, the purified protein was submitted to Isothermal Titration Calorimetry (ITC) binding experiments (**Figure 1**). The derived dissociation constants are reported in **Figure 2** whereas the corresponding changes in enthalpy and Gibbs free energy are reported in Supplementary Table 2.

Prior to all protein titrations, ligands were injected into buffer to determine the corresponding dilution heats (upper traces in **Figure 1**). The titration of the protein with benzoate resulted in exothermic heats that diminished as protein saturation advanced and a *K*_D_ of 90 ± 8 μM was obtained (**Figure 1A**). No binding heats were observed for the linear C6 carboxylate adipate (**Figure 1A**), illustrating the importance of the C6 ring in the molecular recognition. In all experiments where an absence of binding was noted, the protein-ligand mixture was titrated with benzoate to verify protein integrity. In addition, when an absence of binding was noted, the experiment was repeated at a different analysis temperature to verify whether potential endothermic and exothermic contributions may cancel out each other at a given analysis temperature. Subsequent studies were aimed at determining the effect of different benzoate substitutions on binding. To this end, the three isomers of hydroxy-, amino-, methyl-, chloro-, and nitro- benzoates were analyzed (**Figure 2**). The 4-hydroxy substitution of benzoate resulted in an around 12-fold increase in affinity (*K*_D_ = 7.2 μM) as compared to benzoate and was by far the tightest binding mono-substituted benzoate derivative (**Figures 1B**, **2**). In contrast, 4-hydroxybenzaldehyde was devoid of binding (**Figure 1B**) demonstrating the essential role of the carboxyl group for binding. Of the remaining 14 singly substituted benzoates tested, PcaY_PP bound 10 of them with *K*_D_ values between 24 μM for 2-hydroxybenzoate (salicylate) and 138 μM for 3-aminobenzoate (**Figure 2**). Only two 2-substituted derivatives showed binding whereas all five 4-substituted benzoates bound (**Figures 1B,C**, **2**).

Additionally, we analyzed the three doubly substituted benzoates, protocatechuate, vanillate, and vanillin. Interestingly, the former two compounds had a relatively high affinity, with *K*_D_ values of 6.4 and 11 μM, respectively, that were comparable to that of 4-hydroxybenzoate (*K*_D_ = 7.2 μM) (**Figures 1D**, **2**), the tightest binding mono-substituted benzoate. The absence of binding for vanillin underlines the necessity of a carboxyl group for recognition. Finally, the non-aromatic compounds shikimate, quinate and 3-dehydroshikimate were also tested. Surprisingly, from all compounds tested, these non-aromatics showed the highest affinity (**Figures 1D**, **2**). Consequently, PcaY_PP recognizes different aromatic and non-aromatic chemoeffectors directly. However, the receptor recognizes the non-aromatic chemoeffectors with higher affinity. Importantly, of the 24 ligands tested, 75% of them are natural compounds (marked with an asterisk in **Figure 2** and Supplementary Table 3). In addition, recognition of all ligands was driven by favorable enthalpy changes that were counterbalanced by unfavorable entropy changes (Supplementary Table 2).

Aromatic acid responsive chemoreceptors MCP2201 and MCP2983 of *C. testosteroni* are stimulated by the binding of different TCA cycle intermediates (Ni et al., 2013a, 2015). The titration of PcaY_PP-LBD with *cis*-aconitate, succinate and malate did not show any binding (data not shown).

### Ligand Binding Stabilizes the PcaY_PP-LBD Dimer

**FIGURE 3:**
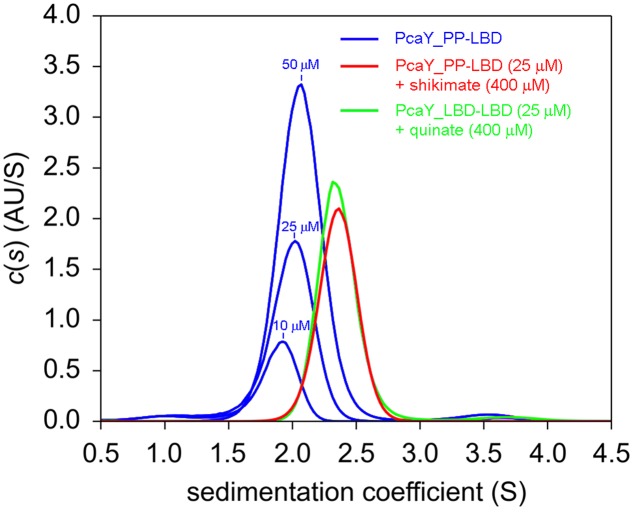
Sedimentation velocity analytical ultracentrifugation studies of PcaY_PP-LBD. Sedimentation coefficient profiles obtained for protein in the absence and presence of ligands.

Four-helix bundle domains are ubiquitous sensor domains in bacterial signal transduction systems (Ulrich and Zhulin, 2005). In order to determine the effect of ligand binding on the oligomeric state of PcaY_PP-LBD, we carried out analytical ultracentrifugation studies (AUC). In initial sedimentation velocity assays sedimentation coefficients of ligand-free PcaY_PP-LBD were determined at three different concentrations (**Figure 3**). Data showed a single species (**Figure 3**) with a sedimentation coefficient that is slightly dependent on the protein concentration. At the intermediate concentration of 25 μM, the corrected standard s_20,w_ is 2.6 S, with a frictional ratio of 1.4. This corresponds to a virtual intermediate species with a molecular weight of 31 kDa, which is between the monomeric and the dimeric species. This virtual species corresponds to rapidly interchanging monomers and dimers and the dimerization-dissociation kinetics is faster than the timescale of the AUC experiment. Similar virtual species for recombinant LBDs have been observed previously (Martin-Mora et al., 2016).

When this analysis was repeated in the presence of saturating concentrations of quinate and shikimate, a single species was observed, but its sedimentation coefficient had shifted to s_20,w_ = 3.1 S, with a frictional ratio of 1.3 (slightly elongated). The molecular weight calculated for this species from the sedimentation coefficient corresponds to 38.0 kDa, which is very close to the sequence derived molecular weight of the PcaY_PP-LBD dimer of 38.4 kDa. It can therefore be concluded that the binding of ligands stabilizes the PcaY_PP-LBD dimer.

**FIGURE 4:**
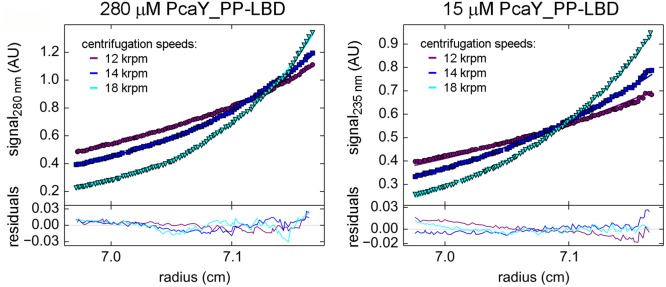
Sedimentation equilibrium analytical ultracentrifugation studies of PcaY_PP-LBD. Absorbance profiles of two different concentrations of ligand-free PcaY_PP-LBD at three different speeds. The experimental values are represented by filled symbols while the line represents the best global fit to these values for the six concentrations, three speeds, and two wavelengths (30 curves) used in this experiment.

The concentration dependence of the sedimentation coefficient of ligand-free PcaY_PP-LBD and the fact that the single peak corresponds to a virtual intermediate species suggest that the protein is present in a monomer-dimer equilibrium. To characterize this equilibrium we conducted a multi-speed and multi-wavelength sedimentation equilibrium experiments covering the protein concentration range of 15 to 280 μM (**Figure 4**). When data were globally fitted to a monomer–dimer model a *K*_d_ = 57.5 μM was obtained for the protein auto-association (**Figure 4**). This value, combined with the observation that binding in ITC was only observed at higher protein concentrations (above 5 μM), indicates that only dimeric PcaY_PP-LBD is able to bind ligands which in turn causes dimer stabilization.

### Different Magnitude of Chemotactic Response to PcaY_PP Ligands

**FIGURE 5:**
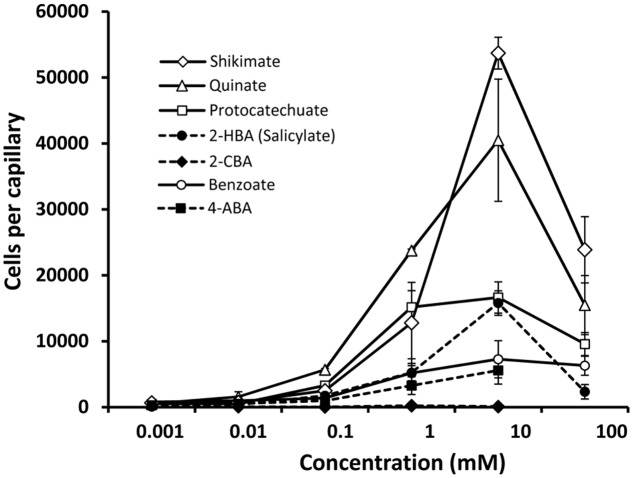
Quantitative capillary chemotaxis assays of *Pseudomonas putida* KT2440 toward different compounds. Data are means and standard deviations from three biological replicates conducted in triplicate. Data were corrected with the number of cells that swam into buffer containing capillaries (1443 ± 370).

Subsequently, we investigated the chemotactic responses of *P. putida* KT2440 to PcaY_PP ligands. To this end, we selected seven compounds that differ in structure and affinity and have conducted quantitative capillary chemotaxis assays (**Figure 5**). Among these compounds was a single compound, 2-chlorobenzoate, that did not bind to PcaY_PP-LBD. As expected this compound did not induce a chemotactic response (**Figure 5**). Relevant chemotaxis was observed for the remaining six compounds at a concentration range between 100 μM to 100 mM, with optimal responses at 10 mM. Interestingly, significant differences were observed in the magnitude of response. Whereas shikimate and quinate produced very strong responses, those of protocatechuate, 2-hydroxybenxoate (salicylate), benzoate and 4-aminobenzoate were much weaker (**Figure 5**). The magnitude of chemotaxis correlated with the *K*_D_ values as determined above (Supplementary Figure 2), indicating that the affinity is a key parameter that defines the magnitude of chemotactic responses.

**FIGURE 6:**
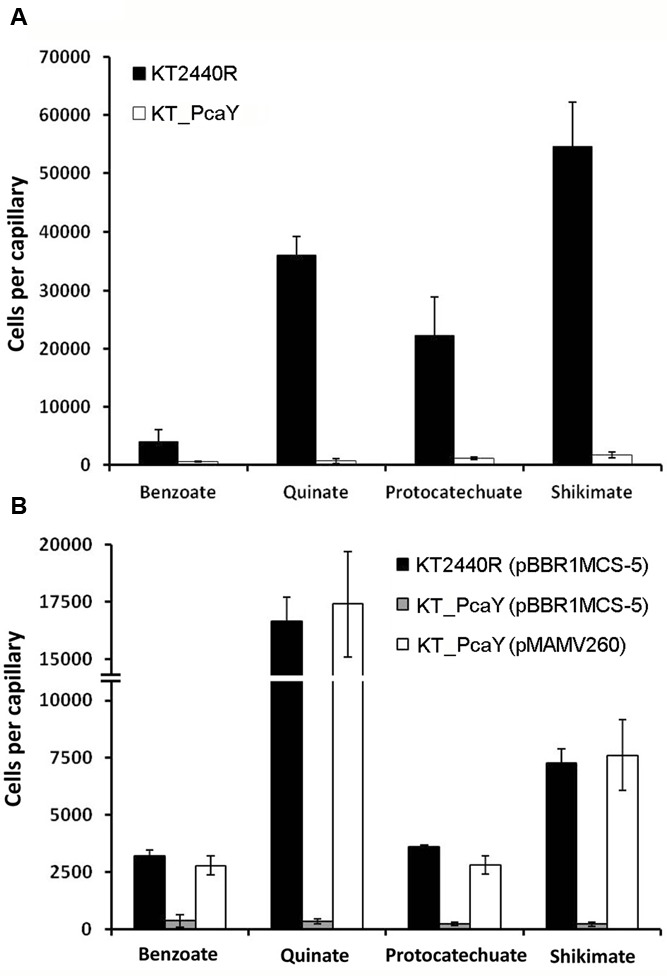
Quantitative capillary chemotaxis assays of *P. putida* KT2440R, its mutant defective in *pcaY_PP* and the complemented mutant strains to different PcaY_PP chemoeffectors. **(A)** Chemotaxis of the wt and its *pcaY_PP* mutant to different chemoeffectors. **(B)** Complementation assays of the *pcaY_PP* mutant by the *in trans* expression of *pcaY_PP* using the pBBRMCS-5-based vector, pMAMV260. As controls, chemotaxis of the wt and mutant strain harboring empty plasmid pBBRMCS-5 were analyzed. The chemoeffector concentration was 10 mM. Data were corrected with the number of cells that swam into buffer containing capillaries (920 ± 105 for **A**; 148 ± 34 for **B**). Data are the means and standard deviations from three biological replicates conducted in triplicate.

To assess the contribution of PcaY_PP to chemotaxis, we characterized a transposon mutant defective in the *pcaY_PP* gene. Initial control experiments involved the assessment of chemotaxis of the wild-type and mutant strain to casamino acids. As shown in Supplementary Figure 3, the inactivation of *pcaY_PP* did not alter amino acid chemotaxis, indicating that the inactivation of this gene did not have any undesired secondary effects. **Figure 6A** illustrates that the inactivation of the *pcaY_PP* gene abolished chemotaxis to the four structurally different chemoattractants studied. To confirm the association between the transposon mutation and the chemotactic phenotype, genetic complementation studies were carried out. For these assays, the *pcaY_PP* gene and its promoter region were cloned into plasmid pBBR1MCS-5 and the resulting pMAMV260 was introduced into the *pcaY_PP* mutant strain. For control purposes, the empty plasmid pBBR1MCS-5 was also introduced into the wt and mutant strains. The resulting strains were submitted to chemotaxis assays to 10 mM solutions of the four selected compounds. Data shown in **Figure 6B** illustrate that the chemotaxis of the complemented mutant was similar to that of the wt strain bearing empty plasmid pBBR1MCS-5. Taken together, data indicate that PcaY_PP is the physiologically relevant receptor for these compounds in *P. putida* KT2440.

### Inactivation of *pcaY_PP* Does Not Affect Root Colonization

For root-associated bacteria, chemotaxis to plant root exudates is an essential prerequisite for efficient root colonization (Scharf et al., 2016). We have shown recently that the mutation of *mcpG* and *mcpU*, encoding chemoreceptors for gamma-aminobutyrate and polyamines, respectively, reduced plant root colonization capabilities by *P. putida* KT2440 (Reyes-Darias et al., 2015a; Corral-Lugo et al., 2016). The composition of root exudates is very complex and strong attractants such as quinate, shikimate, salicylate, or benzoate were identified in plant root exudates (Khorassani et al., 2011; Tawaraya et al., 2014; Bowsher et al., 2016). To investigate the implications of PcaY_PP in the efficient colonization of the rhizosphere, we conducted competitive root colonization studies. In these assays, maize roots were inoculated with 1:1 mixtures of the wt and *pcaY_PP* mutant strain. After 7 days, bacteria were recovered from the rhizosphere and the root tip for quantification. As shown in Supplementary Figure 4, the ratios of recovered wt to mutant cells were approximately 1:1, indicating that *pcaY_PP* inactivation did not alter competitive plant root colonization.

### Only Seven of the Ligands Identified Support Bacterial Growth As Sole Carbon Source

**FIGURE 7:**
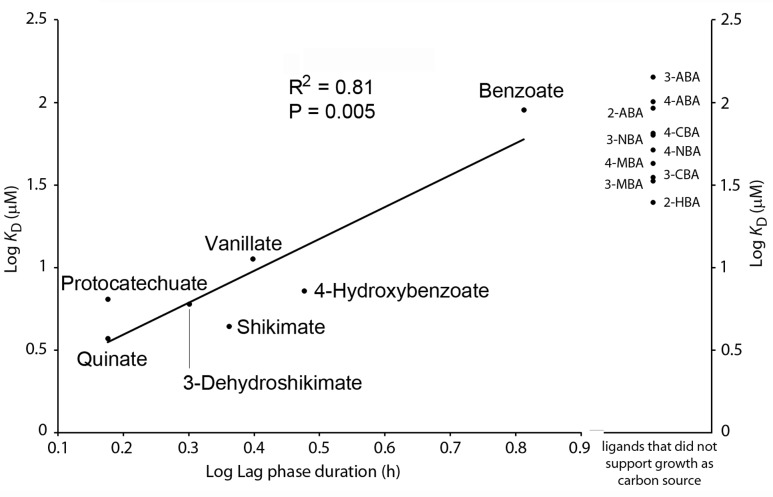
Relationship between binding affinity of chemoeffectors to PcaY_PP-LBD and the capacity of these compounds to support bacterial growth as sole C-source. Left: Plot of the dissociation constants as determined by ITC (see **Figure 2**) and the duration of the lag time during growth on minimal medium containing the compound indicated a sole carbon source. Data were fitted by linear regression. Right: Dissociation constants obtained for PcaY_PP ligands that did not support growth as sole carbon source.

Binding studies have resulted in the identification of 17 ligands recognized by PcaY_PP. To assess which of these compounds are able to sustain growth of *P. putida* KT2440 as sole carbon source, we conducted growth experiments in M9 minimal medium supplemented with each of the PcaY_PP ligands. Only seven ligands supported bacterial growth, which were benzoate, 4-hydroxybenzoate, protocatechuate, vanillate, quinate, shikimate, and dehydroshikimate (gray shading in **Figure 2**). Of the 15 singly substituted benzoate derivatives, only a single compound, 4-hydroxybenzoate, supported growth. Interestingly, the affinity of this compound was significantly higher than those of all other singly substituted benzoates (**Figure 2**). As exemplified in Supplementary Figure 5 for quinate and benzoate, there were significant differences in the growth kinetics. From these growth experiments, we have derived bacterial yields as well as generation- and lag times. In subsequent analyses, we determined whether the chemoeffector affinity as determined by ITC correlates with any of these parameters. As shown in Supplementary Figure 6 no correlation was observed between the affinity and the yield or generation time. In contrast, differences observed in the lag phase of bacterial cultures correlated with the chemoeffector *K*_D_ for the chemoreceptor (**Figure 7**). In a second analysis, we introduced the *K*_D_ values of the 10 PcaY_PP ligands that did not support into the graph of **Figure 7**. These compounds grouped together in the weaker affinity range. These data were submitted to a χ^2^ test for a two-category statistical analysis defining the categories as ligands that can or cannot be used as C-sources and compounds having high (*K*_D_ < 20 μM) and low (*K*_D_ > 20 μM) affinity. A χ^2^ of 3500 and a corresponding *P*-value of 0.03 was obtained indicating a correlation between the chemoeffector affinity for its chemoreceptor and the capacity to sustain growth as sole C-source. Taken together, there are several pieces of evidence indicating a relationship between the metabolic value of compounds and their recognition by the PcaY_PP chemoreceptor.

## Discussion

In the model organism of this study, *P. putida* KT2440, eight chemoreceptors have been functionally characterized (Supplementary Table 1). These receptors were shown to be activated by the direct binding of 34 different chemoeffectors, which are all of metabolic value since they support growth as C- and/or N-sources. The PcaY_PP receptor seems to be different since only some of the identified ligands supported growth as sole C-source (**Figures 2**, **7**). As illustrated in **Figure 5**, ligands of both classes cause chemoattraction.

Chemotactic signaling is decoupled from uptake and metabolism of compounds. We report here several pieces of evidence suggesting that the metabolic capacity of the chemoeffectors is an important parameter in the recognition of ligands by PcaY_PP. Firstly, there was a correlation between the duration of the growth lag time and the dissociation constant (**Figure 7**). Secondly, the *K*_D_ values of non-metabolizable ligands were significantly higher than those of metabolizable ligands (**Figures 2**, **7**). Thirdly, of the 15 singly substituted benzoate derivatives, there was only one, 4-hydroxybenzoate, that served for growth. The *K*_D_ value of this compound (7.2 ± 1 μM) was significantly lower than that of all other singly substituted benzoates, which had an average *K*_D_ of 63 ± 35 μM. Striking parallels exist to a study that evaluated the relationship between chemotaxis and metabolism to amino acids, which are among the most valuable nutrient sources for bacteria (Yang et al., 2015). In *E. coli* there was a nearly perfect overlap between chemoattractive and catabolized amino acids. Seven amino acids were identified as specific chemoattractants, of which six were rapidly consumed, whereas only one amino acid that served for growth was not an attractant. In addition, a second correlation was observed between the order of amino acid utilization and their efficiency as chemoattractants. The authors conclude that the chemotaxis system of *E. coli* has evolved to specifically locate sources of most nutritionally valuable amino acids (Yang et al., 2015). In marked contrast no such correlations were reported for *Bacillus subtilis* and the authors speculated that *B. subtilis* rather uses gradients of amino acids as general cues to locate sources of nutrients such as plant roots or to initiate symbiosis or pathogenesis. Our data indicate a similar chemotaxis-metabolism relationship as observed for *E. coli*. Data reported furthermore indicate that the modulation of the binding affinity at the chemoreceptor is a mechanism by which the metabolic potential of compounds reflects on the magnitude of chemotaxis.

The dissociation constants of different ligands to the purified LBDs of all characterized *P. putida* KT2440 chemoreceptors are shown in Supplementary Table 4. All constants were determined using isothermal titration calorimetry using very similar experimental conditions. Of these receptors only one binds specifically one compound, namely the gamma-aminobutyrate (GABA) specific McpG (Reyes-Darias et al., 2015a), whereas all other receptors recognize multiple ligands. Among all receptors, GABA binding at McpG-LBD showed the highest affinity (*K*_D_ = 0.17 μM), which is consistent with the notion that ligand recognition is a trade-off between affinity and specificity. The dissociation constants of the PcaY_PP-LBD ligands, as determined here, are in the same range as the constants for the remaining characterized *P. putida* KT2440 receptors (Supplementary Table 4). The data reported also reveal the minimal ligand requirements for binding, which consist in a 6-membered carbon ring and a carboxylic acid group. For the recognition of aromatic compounds, the substitution at the C2-position was an essential criteria (only hydroxyl and amino substituents bound), whereas the nature of substitutions at the C3- and C4-positions were less critical (**Figure 2**). In this context interesting parallels exist to the broad range aromatics sensing TodS histidine kinase, where C2-substituents determined largely the magnitude of regulatory response (Busch et al., 2007).

PcaY (Luu et al., 2015) and PcaY_PP share a highly similar ligand spectrum and in both cases a significant number of chemoeffectors were found to lack metabolic value. The questions that remained unanswered in Luu et al. (2015) were, firstly, whether the chemoeffectors identified or metabolic derivatives thereof, like in the case of the *C. testosteroni* aromatic acid receptors (Ni et al., 2013a, 2015), activated the receptor and, secondly, whether this activation occurred via direct binding or an alternative indirect mechanism. We show here that receptor activation is achieved through the direct binding of chemoeffectors to the LBD of the receptor. Another question concerns the potential physiological benefit of chemotaxis to compounds that are of no apparent value. So far no answer can be given to this question, but data on PcaY and PcaY_PP show clear parallels to the recognition of aromatic compounds by other bacterial sensor proteins. Thus, due to the action of the toluene dioxygenase (TOD) pathway, *P. putida* DOT-T1E can metabolize benzene, toluene and ethylbenzene and use these compounds as sole carbon sources. However, the McpT chemoreceptor of this strain mediates chemotaxis, apart from these three TOD substrates, to an extensive array of structurally related but non-metabolizable mono- and biaromatic compounds (Lacal et al., 2011). Very similar observations have been made in the study of the TodS sensor kinase that controls the expression of the TOD pathway operon. Apart from the three substrates, TodS recognizes many different non-metabolizable aromatic compounds and induces pathway expression in their response (Lacal et al., 2006; Busch et al., 2007). Taken together, it can be speculated that these chemotactic and transcriptional responses to compounds that lack apparent metabolic value may be related to the molecular complexity of evolving molecular recognition mechanism that are specific for certain aromatic compounds. As a consequence, some sensor proteins may have evolved to recognize many different ligands of which only some are of metabolic value. It is plausible that of compounds without any apparent metabolic value may represent environmental cues and their detection may be beneficial to the bacterium as proposed by (Yang et al., 2015).

**FIGURE 8:**
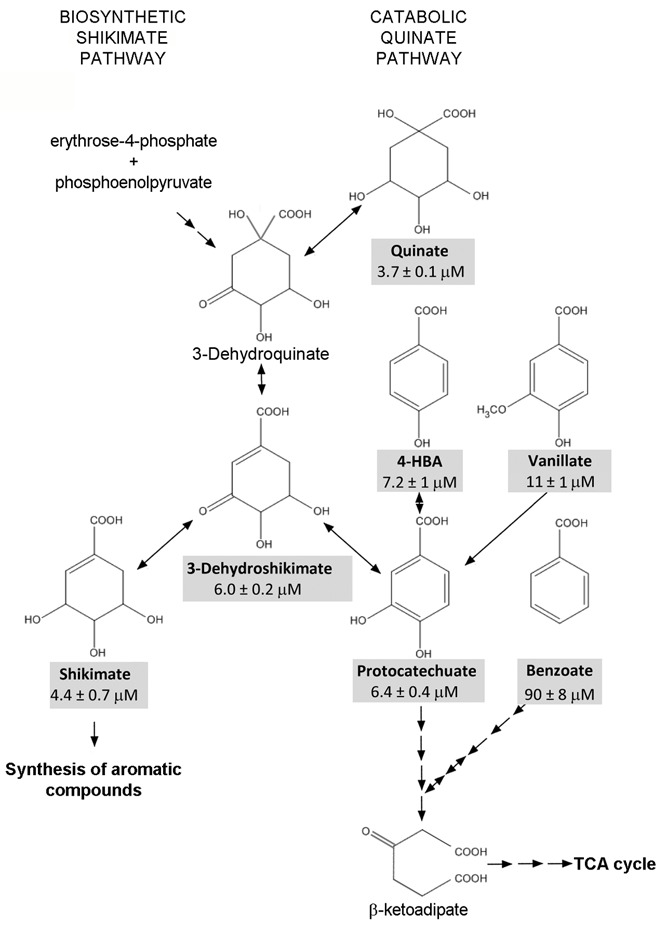
Schematic view of metabolic pathways containing the metabolizable PcaY_PP ligands. Compounds shaded in gray are PcaY_PP ligands. Information taken from the metabolic map of *P. putida* KT2440 in the KEGG database (Kanehisa et al., 2017).

We show here that the chemoeffector affinity for PcaY_PP determines the magnitude of the chemotactic response (Supplementary Figure 2), which is a correlation that has already observed for other broad range chemoreceptors (Reyes-Darias et al., 2015b) but that does not apply to all chemoreceptors (Lacal et al., 2010). The tightest binding ligand was quinate, followed by shikimate, 3-dehydroshikimate and protocatechuate. These compounds are located at the intersection of two metabolic pathways, namely the catabolic quinate pathway that feeds into the β-ketoadipate pathway and subsequently into the TCA cycle, and the shikimate pathway for the biosynthesis of most aromatic compounds (**Figure 8**). In fact, 3-dehydroshikimate is an intermediate of both pathways. Chemotaxis to these ligands is therefore of double benefit since it permits access to C- and energy sources as well as to compounds that are required for the biosynthesis of aromatic compounds. Parallels exist to other chemoreceptors where receptor ligands are equally at the cross-roads of catabolic and biosynthetic pathways. One such example is the McpH chemoreceptor that is responsible for chemotaxis to adenine and guanine (Fernandez et al., 2016). Both compounds are at the beginning of the purine degradation pathway for their conversion into urea which can be used as sole N-source. Both bases, however, also feed into the biosynthetic pathway of purine nucleotides. In the particular case of quinate, this cyclohexanecarboxylic acid is abundantly present in many different plants (Bentley, 1990; Lehmann et al., 2016) and was shown to accumulate to concentrations above 50% of the plant dry weight (Bhatia et al., 2015), suggesting that it may serve as carbon storage compound. It is therefore not astonishing that a plant saprophyte like *P. putida* KT2440 has a high-affinity chemoreceptor for quinate.

Several studies show that *P. putida* KT2440 is able to efficiently colonize the rhizosphere in non-sterilized soils, i.e., in the presence of naturally occurring soil bacteria (Molina et al., 2000; Matilla et al., 2010; Neal et al., 2012; Pizarro-Tobias et al., 2015). In addition, competition assays with other *P. putida*, *P. fluorescens*, and *P. chlororaphis* strains show that KT2440 is equally efficient in rhizosphere colonization (Molina et al., 2000). Chemotaxis was found to be essential for efficient root colonization (Scharf et al., 2016). Although the PcaY_PP chemoreceptor appears to be dispensable for efficient root colonization (Supplementary Figure 4), other KT2440 chemoreceptors like the GABA specific McpG (Reyes-Darias et al., 2015a) and the polyamine specific McpU (Corral-Lugo et al., 2016) were found to be necessary for optimal root colonization.

Similar to KT2440, *C. testosteroni* CNB-1 shows chemoattraction to aromatic acids like benzoate, 3-hydroxybenzoate, 4-hydroxybenzoate, protocatechuate, vanillin, or vanillate (Ni et al., 2013a). All of these compounds support the growth of the bacterium (Ni et al., 2013b) and further studies showed that the chemoreceptors MCP2201, MCP2901, and MCP2983 mediate taxis to these compounds (Ni et al., 2013a, 2015; Huang et al., 2016). These receptors possess a four-helix bundle LBD but employ different mechanisms. Surprisingly, the recombinant LBDs of receptors MCP2201 and MCP2983 did not bind any of the aromatic acids. Instead, MCP2201-LBD bound a series of TCA cycle intermediates (Ni et al., 2013a), whereas MCP2983 recognized *cis*-aconitate (Ni et al., 2015). These compounds are generated by the metabolism of aromatic acids and it was proposed that MCP2201- and MCP2983-mediated chemotaxis to aromatic acids is caused by the recognition of the resulting metabolites (Ni et al., 2013a). This hypothesis was supported by the fact that the observed chemotaxis was metabolism dependent (Ni et al., 2013a). In marked contrast, the mechanism of MCP2901 is based on the direct binding of aromatic acids to the periplasmic LBD (Huang et al., 2016); although affinities were relatively weak (*K*_D_ values typically of several hundreds of μM) and thus inferior to the corresponding values of PcaY_PP. Taken together, there are thus two fundamentally different mechanism of chemotaxis to aromatic acids. One mechanism (MCP2201 and MCP2983) is based on the recognition of TCA cycle intermediates that are generated by the aromatic acid metabolism, whereas the mechanism of MCP2901 and PcaY_PP relies on the direct recognition of aromatic acids by periplasmic LBDs.

The Tar receptor is the primary model in the study of chemoreceptors. Classical biochemical studies showed that the recombinant Tar-LBD is present in a monomer-dimer equilibrium and the self-association constant was estimated to be in between 0.5 and 5 μM. Aspartate binding was shown to increase the dimer formation and it was estimated that aspartate causes a decrease of the dimer self-association constant by a factor of around 100 (Milligan and Koshland, 1993). These data have prompted the hypothesis that ligand induced receptor dimerization is a prerequisite for signaling (Stock, 1996). Using AUC techniques we have assessed this issue for PcaY_PP. In the absence of ligands, sedimentation velocity studies showed that the protein is present as a virtual species formed by rapidly interchanging monomers and dimers. Multi-speed and multi-wavelength sedimentation equilibrium experiments have resulted in the calculation of the dimer association constant of 57.5 μM. In the presence of ligands, the protein was exclusively present as dimer, confirming the data obtained for Tar-LBD. Ligand induced LBD-dimer stabilization has also been observed for the 4HB domain containing receptor CtpH (Rico-Jimenez et al., 2016) as well as for receptors containing a helical bimodular domain (Lacal et al., 2010; Martin-Mora et al., 2016). Inspection of four-helix bundle and helical bimodular domain structures revealed the molecular basis for this stabilization since ligands bind at the dimer interface and amino acids from both monomers establish direct contacts with bound ligand (Milburn et al., 1991; Pineda-Molina et al., 2012). This, however, does not appear to be the case for all chemoreceptor domains since, for example, dCACHE domains were found to be monomeric in solution in the presence and absence of ligand (Rico-Jimenez et al., 2013) and structural information showed that amino acids from only one monomer establish contacts with the bound ligand (Liu et al., 2015). Significant progress has been made in the chemoreceptor identification by high-throughput based ligand screening of recombinant LBDs (McKellar et al., 2015; Fernandez et al., 2016). The monomer-dimer issue is central to the success of this approach since, in the case of four-helix bundle and helical bimodular domains, it has to be assured that at least a fraction of the protein is in a dimeric state.

## Author Contributions

TK conceived and coordinated the study and wrote the paper. MF and MM designed, performed and analyzed the experiments shown in **Figures 1**, **2**, **5**–**7**. AO designed, performed and analyzed the experiments shown in **Figures 3**, **4**. All authors reviewed the results and approved the final version of the manuscript.

## Conflict of Interest Statement

The authors declare that the research was conducted in the absence of any commercial or financial relationships that could be construed as a potential conflict of interest. The reviewer FAP and handling Editor declared their shared affiliation, and the handling Editor states that the process nevertheless met the standards of a fair and objective review.
