# Supplementary material for: Metabolic Value Chemoattractants Are Preferentially Recognized at Broad Ligand Range Chemoreceptor of Pseudomonas putida KT2440
Source: Front Microbiol. 2017 May 31;8:990. doi: 10.3389/fmicb.2017.00990 (PMC5449446; doi:10.3389/fmicb.2017.00990)
Supplement: Supplementary file 1 [file Data_Sheet_1.docx]

Supplementary material

to

**Metabolic value chemoattractants are preferentially recognized at broad ligand range chemoreceptor of *Pseudomonas putida* KT2440**

by

Matilde Fernández, Miguel A. Matilla, Álvaro Ortega and Tino Krell


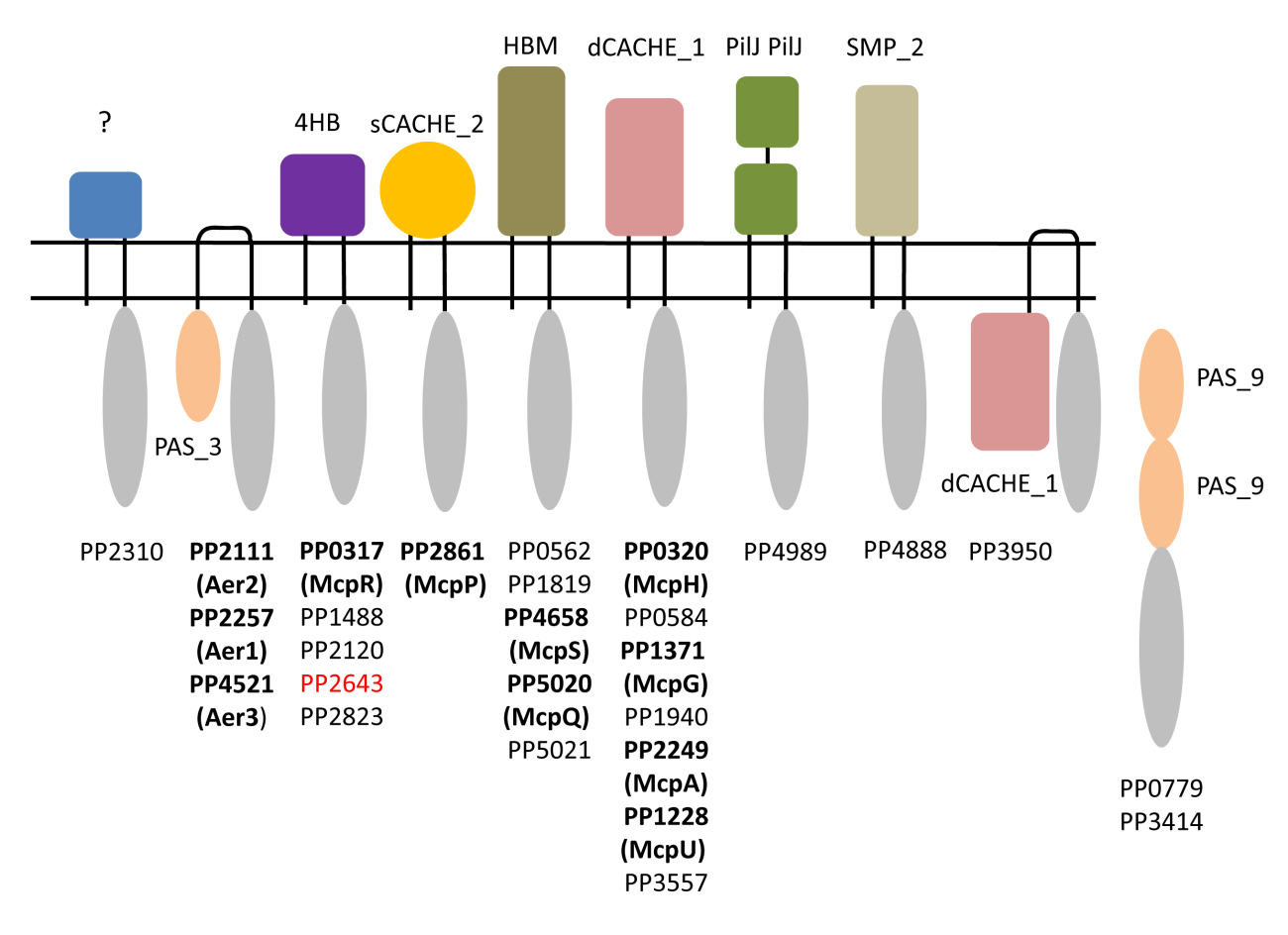


**Supp. Fig. 1) Schematic view of the topology and domain composition of chemoreceptors from *P. putida* KT2440.** Functionally annotated receptor are highlighted in bold (for further information refer to Supp. Table 1) and the chemoreceptor under investigation is shown in red. PAS: Per-Arnt-Sim domain, 4HB: 4-Helix Bundle domain, sCACHE: single Calcium and Chemotaxis receptor domain; HBM: Helical Bimodular domain, dCACHE_1: double Calcium and Chemotaxis receptor domain, PilJ: Type IV pili methyl-accepting chemotaxis transducer N-terminal domain; SMP_2: Bacterial virulence factor haemolysin domain.

**

**

**Supp. Fig. 2) Plot of the logarithm of the dissociation constants of different chemoeffectors as determined by Isothermal Titration Calorimetry against the logarithms of the magnitude of chemotaxis for 1 (A) and 10 mM (B) of chemoeffectors.** Chemotaxis data are shown in Fig. 5. The lines are fits by a least squares linear regression and in both cases the resulting deviations from zero were significant.

**
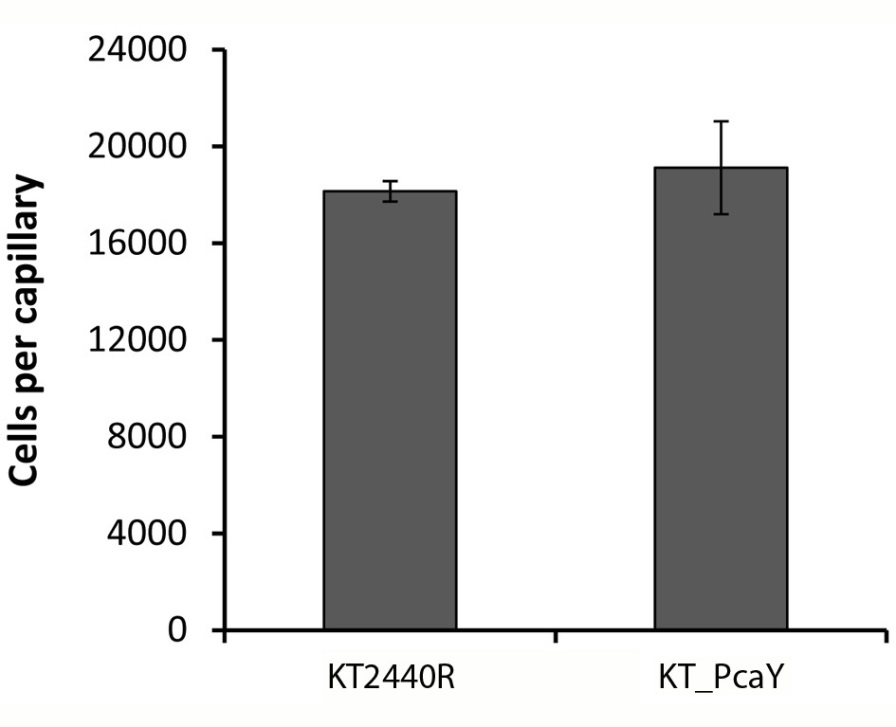
**

**Supp. Fig. 3) Quantitative capillary chemotaxis assays of *P. putida* KT2440R and its mutant in the *pcaY_PP* gene towards 0.1 % (w/v) casamino acids.** Data were corrected with the number of cells that swam into buffer containing capillaries (413 ± 61). Data are the means and standard deviations from three biological replicates conducted in triplicate.

**
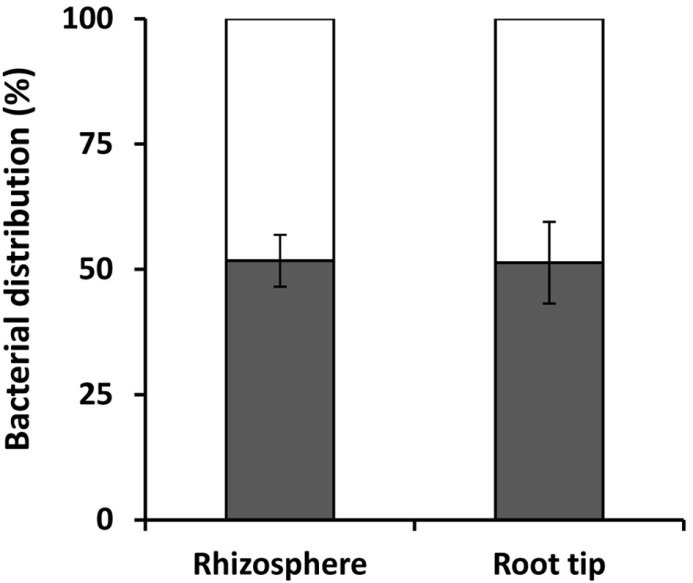
**

**Supp. Fig. 4) Root colonization assays of *P. putida* KT2440R and its *pcaY_PP* mutant.** The figure represents the percentage of KT2440RTn*7*-Sm (grey) and the mutant in the *pcaY_PP* gene (white) recovered from the rhizosphere and root tips of maize (*Zea mays*) plants 7 days after inoculation. Data are the means and standard deviations of 6 plants.


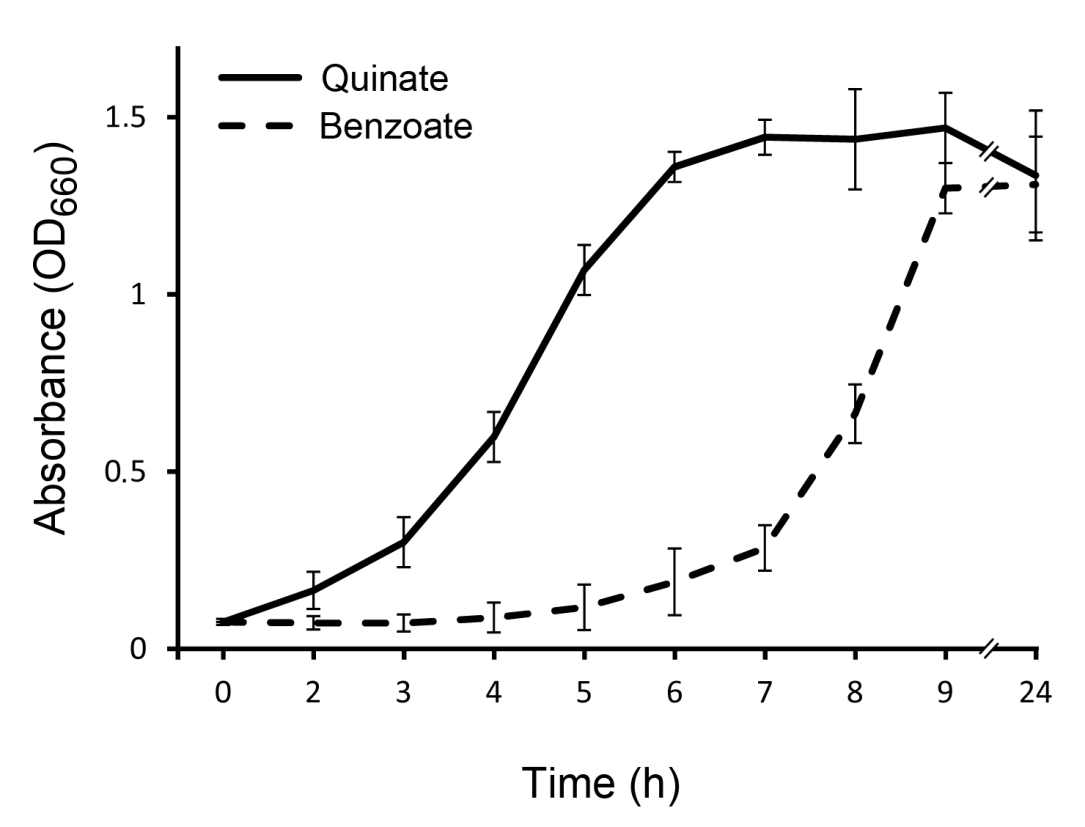


**Supp. Fig. 5) Growth of *P. putida* KT2440 on M9 minimal medium supplemented with 10 mM of quinate or benzoate.** Data are means and standard errors of three biological replicates.





**Supp. Fig. 6) Plot of the logarithm of the dissociation constants of different chemoeffectors as determined by Isothermal Titration Calorimetry against the logarithms of the bacterial yield and generation time as derived from growth experiments in M9 minimal medium supplemented with each of these ligands.** The lines are fits by a least squares linear regression and in both cases the resulting deviations from zero were not significant.

**Supp. Table 1) Summary of information available on *P. putida* KT2440 chemoreceptors.**

| Code (name) | chemoeffector | references | comment |
| --- | --- | --- | --- |
| PP_0317 (McpR) | succinate, malate, fumarate | (1) | effectors identified *in vivo* |
| PP_0320 (McpH) | adenine, guanine, hypoxanthine, xanthine, uric acid, purine | (2) | specific for metabolizable purines |
| PP_1228 (McpU) | putrescine, spermidine, cadaverine | (3) | specific for polyamines |
| PP_1371 (McpG) | GABA | (4) | binds GABA exclusively |
| PP_1488 | unknown | (3) | homologue of *P. aeruginosa* WspA, |
| PP_2111 (Aer2) | energy taxis | (5) |  |
| PP_2249 (McpA) | Gly, L-isomers of Ala, Cys, Ser, Asn, Gln, Phe, Tyr, Val, Ile, Met, Arg | (3) | specific for the L-isomers |
| PP_2257 (Aer1) | energy taxis? | (5) |  |
| PP_2861 (McpP) | pyruvate, L-lactate, propionate, acetate | (6) |  |
| PP_4521 (Aer3) | energy taxis? | (5) |  |
| PP_4658 (McpS) | malate, fumarate, oxaloacetate, succinate, citrate, isocitrate, butyrate | (7-9) | does not bind citrate/ metal^2+^ complexes |
| PP_5020 (McpQ) | citrate, citrate/metal^2+^ | (10) | specific for citrate |
| PP_0562 | inorganic phosphate (by homology) | (12) | homologue of *P. aeruginosa* CtpL |
| PP_0584 | unknown |  |  |
| PP_0779 | unknown |  |  |
| PP_1819 | unknown |  |  |
| PP_1940 | unknown |  |  |
| PP_2120 | inorganic phosphate (by homology) | (11) | homologue of *P. aeruginosa* CtpH |
| PP_2310 | unknown | (3) | mutation changes biofilm formation |
| PP_2643 (PcaY_PP) | different cyclic acids | (12) | homologue of *P. putida* F1 PcaY |
| PP_2823 | unknown |  |  |
| PP_3414 | unknown |  |  |
| PP_3557 | unknown |  |  |
| PP_3950 | unknown |  |  |
| PP_4888 | unknown | (13) | DIMBOA increases receptor expression |
| PP_4989 | unknown | (14) | homologue of *P. aeruginosa* PilJ |
| PP_5021 | unknown |  |  |

**Supp. Table 2) Changes in enthalpy and Gibbs free energy as derived from isothermal titration calorimetry studies of ligand recognition by PcaY_PP-LBD.**

| **Compound** | **∆*H* (kcal/mol)** | **∆*G* (kcal/mol)** |
| --- | --- | --- |
| Quinate | -13.2 ± 0.4 | -7.40 ± 0.02 |
| Shikimate | -16.6 ± 1.1 | -7.30 ± 0.1 |
| 3-Dehydroshikimate | -19.9 ± 0.3 | -7.11 ± 0.03 |
| Protocatechuate | -22.9 ± 1.2 | -7.08 ± 0.04 |
| Benzoate | -13.3 ± 0.5 | -5.51 ± 0.06 |
| 2-Hydroxybenzoate | -21.0 ± 0.6 | -6.30 ± 0.03 |
| 4-Hydroxybenzoate | -33.2 ± 1.4 | -7.01 ± 0.08 |
| Vanillate | -12.8 ± 1.7 | -6.70 ± 0.03 |
| 2-Aminobenzoate | -18.0 ± 1.8 | -5.52 ± 0.07 |
| 3-Aminobenzoate | -16.4 ± 1.2 | -5.26 ± 0.07 |
| 4-Aminobenzoate | -11.0 ± 0.4 | -5.46 ± 0.12 |
| 3-Chlorobenzoate | -13.2 ± 0.3 | -6.09 ± 0.03 |
| 4-Chlorobenzoate | -13.0 ± 0.3 | -5.72 ± 0.05 |
| 3-Nitrobenzoate | -17.5 ± 0.3 | -5.74 ± 0.06 |
| 4-Nitrobenzoate | -13.8 ± 1.2 | -5.86 ± 0.06 |
| 3-Methylbenzoate | -21.9 ± 1.1 | -6.12 ± 0.04 |
| 4-Methylbenzoate | -13.2 ± 0.9 | -5.97 ± 0.12 |

**Supp. Table 3) Natural or non-natural occurrence of ligands tested.** Data were retrieved from the Zinc database (<http://zinc.docking.org/>) of compounds (15). Further information on these compounds can be obtained by entering the code into the Zinc database.

| **Compound Natural/Non-natural code Zinc database** |
| --- |
|  |
| Benzoate Natural ZINC1011 |
| 2-HBA Natural ZINC1554 |
| 2-ABA Natural (Vitamin L) ZINC47985 |
| 2-MBA Natural ZINC1850420 |
| 2-CBA Non-natural ZINC330133 |
| 2-NBA Non-natural ZINC80841 |
| 3-HBA Natural ZINC388754 |
| 3-ABA Natural (Gabaculine) ZINC388179 |
| 3-MBA Non-natural ZINC330142 |
| 3-CBA Natural ZINC156863 |
| 3-NBA Non-natural ZINC156875 |
| 4-Hbenzaldehyde Natural ZINC156709 |
| 4-HBA Natural (Paraben) ZINC332752 |
| 4-ABA Natural (Pab) ZINC920 |
| 4-MBA Natural ZINC330134 |
| 4-CBA Non-natural ZINC156865 |
| 4-NBA Non-natural ZINC1688307 |
| Protocatechuate Natural ZINC13246 |
| Vanillate Natural ZINC338275 |
| Vanillin Natural ZINC2567933 |
| Quinate Natural ZINC100009542 |
| Shikimate Natural ZINC3860720 |
| 3-Dehydroshikimate Natural ZINC100018238 |
| Adipate Natural ZINC1530348 |

**Supp. Table 4) Summary of dissociation constants determined by isothermal titration calorimetry binding experiments of different ligands to the purified recombinant ligand binding domains of chemoreceptors from *P. putida* KT2440.**

| Receptor | ligand | *K*_D_  (µM) | Ref. |
| --- | --- | --- | --- |
| PcaY_PP | Benzoate  2-HBA  2-ABA  3-ABA  3-MBA  3-CBA  3-NBA  4-HBA  4-ABA  4-MBA  4-CBA  4-NBA  Protocatechuate  Vanillate  Quinate  Shikimate  Dehydroshikimate | 90  24  89  138  32  34  61  7.2  98  41  64  50  6.4  11  3.7  4.4  6 | This work |
| McpS | Succinate  Fumarate  Malate  Oxalacetate  Citrate  Isocitrate  Butyrate  Acetate | 82  17  8.4  24  109  337  92  574 | (7-9) |
| McpQ  Receptor | Citrate  Citrate/Mg^2+^  Citrate/Ca^2+^  Ligand | 39  27  14  *K*_D_  (µM) | (10)  Ref. |
| McpU | Putrescine  Cadaverine  Spermidine | 2  22  4.5 | (3) |
| McpA | Gly  L-Ala  L-Cys  L-Ser  L-Asn  L-Gln  L-Phe  L-Tyr  L-Val  L-Ile  L-Met  L-Arg | 35  13  0.6  43  4.3  5.5  2.3  12.1  373  85  5.8  1.2 | (3) |
| McpH | Adenine  Guanine  Xanthine  Hypoxanthine  Purine  Uric acid | 2.4  4.3  2.7  3.3  2.4  1.3 | (2) |
| McpP | Acetate  Propionate  Pyruvate  L-lactate | 34  34  39  107 | (6) |
| McpG | γ-aminobutyrate | 0.17 | (4) |

**References**

1. Parales, R. E., Luu, R. A., Chen, G. Y., Liu, X., Wu, V., Lin, P., Hughes, J. G., Nesteryuk, V., Parales, J. V., and Ditty, J. L. (2013) Pseudomonas putida F1 has multiple chemoreceptors with overlapping specificity for organic acids. *Microbiology* **159**, 1086-1096

2. Fernandez, M., Morel, B., Corral-Lugo, A., and Krell, T. (2016) Identification of a chemoreceptor that specifically mediates chemotaxis toward metabolizable purine derivatives. *Mol Microbiol* **99**, 34-42

3. Corral-Lugo, A., de la Torre, J., Matilla, M. A., Fernandez, M., Morel, B., Espinosa-Urgel, M., and Krell, T. (2016) Assessment of the contribution of chemoreceptor-based signaling to biofilm formation. *Environmental microbiology* **18**, 3355-3372

4. Reyes-Darias, J. A., Garcia, V., Rico-Jimenez, M., Corral-Lugo, A., Lesouhaitier, O., Juarez-Hernandez, D., Yang, Y., Bi, S., Feuilloley, M., Munoz-Rojas, J., Sourjik, V., and Krell, T. (2015) Specific gamma-aminobutyrate chemotaxis in pseudomonads with different lifestyle. *Mol Microbiol* **97**, 488-501

5. Sarand, I., Osterberg, S., Holmqvist, S., Holmfeldt, P., Skarfstad, E., Parales, R. E., and Shingler, V. (2008) Metabolism-dependent taxis towards (methyl)phenols is coupled through the most abundant of three polar localized Aer-like proteins of Pseudomonas putida. *Environmental microbiology* **10**, 1320-1334

6. Garcia, V., Reyes-Darias, J. A., Martin-Mora, D., Morel, B., Matilla, M. A., and Krell, T. (2015) Identification of a Chemoreceptor for C2 and C3 Carboxylic Acids. *Applied and environmental microbiology* **81**, 5449-5457

7. Lacal, J., Alfonso, C., Liu, X., Parales, R. E., Morel, B., Conejero-Lara, F., Rivas, G., Duque, E., Ramos, J. L., and Krell, T. (2010) Identification of a chemoreceptor for tricarboxylic acid cycle intermediates: differential chemotactic response towards receptor ligands. *J Biol Chem* **285**, 23126-23136

8. Lacal, J., Garcia-Fontana, C., Callejo-Garcia, C., Ramos, J. L., and Krell, T. (2011) Physiologically relevant divalent cations modulate citrate recognition by the McpS chemoreceptor. *Journal of molecular recognition : JMR* **24**, 378-385

9. Pineda-Molina, E., Reyes-Darias, J. A., Lacal, J., Ramos, J. L., Garcia-Ruiz, J. M., Gavira, J. A., and Krell, T. (2012) Evidence for chemoreceptors with bimodular ligand-binding regions harboring two signal-binding sites. *Proc Natl Acad Sci U S A* **109**, 18926-18931

10. Martin-Mora, D., Reyes-Darias, J. A., Ortega, A., Corral-Lugo, A., Matilla, M. A., and Krell, T. (2016) McpQ is a specific citrate chemoreceptor that responds preferentially to citrate/metal ion complexes. *Environmental microbiology* **18**, 3284-3295

11. Wu, H., Kato, J., Kuroda, A., Ikeda, T., Takiguchi, N., and Ohtake, H. (2000) Identification and characterization of two chemotactic transducers for inorganic phosphate in Pseudomonas aeruginosa. *J Bacteriol* **182**, 3400-3404

12. Luu, R. A., Kootstra, J. D., Nesteryuk, V., Brunton, C. N., Parales, J. V., Ditty, J. L., and Parales, R. E. (2015) Integration of chemotaxis, transport and catabolism in Pseudomonas putida and identification of the aromatic acid chemoreceptor PcaY. *Mol Microbiol* **96**, 134-147

13. Neal, A. L., Ahmad, S., Gordon-Weeks, R., and Ton, J. (2012) Benzoxazinoids in root exudates of maize attract Pseudomonas putida to the rhizosphere. *PloS one* **7**, e35498

14. Whitchurch, C. B., Leech, A. J., Young, M. D., Kennedy, D., Sargent, J. L., Bertrand, J. J., Semmler, A. B., Mellick, A. S., Martin, P. R., Alm, R. A., Hobbs, M., Beatson, S. A., Huang, B., Nguyen, L., Commolli, J. C., Engel, J. N., Darzins, A., and Mattick, J. S. (2004) Characterization of a complex chemosensory signal transduction system which controls twitching motility in Pseudomonas aeruginosa. *Mol Microbiol* **52**, 873-893

15. Irwin, J. J., Sterling, T., Mysinger, M. M., Bolstad, E. S., and Coleman, R. G. (2012) ZINC: a free tool to discover chemistry for biology. *Journal of chemical information and modeling* **52**, 1757-1768
